# Supplementary material for: Polysarcosine-Functionalized mRNA Lipid Nanoparticles Tailored for Immunotherapy
Source: Pharmaceutics. 2023 Aug 1;15(8):2068. doi: 10.3390/pharmaceutics15082068 (PMC10458461; doi:10.3390/pharmaceutics15082068)
Supplement: Supplementary file 1 [file pharmaceutics-15-02068-s001.zip › Supplementary Materials.pdf]

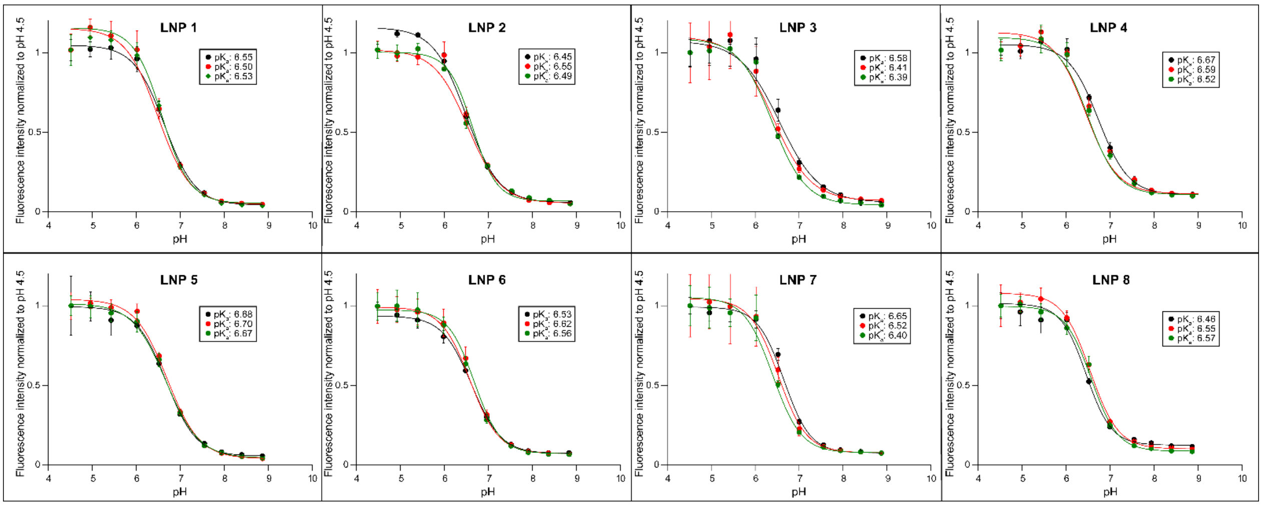

Figure S1: Fluorescence-based assay for apparent pK$_a$ determination. Fluorescence intensity was normalized against the intensity measured at pH 4.5 reflecting a fully protonated state of the ionizable lipid. Boltzmann fitting was utilized to determine the inflection point of each measurement. Data are presented as mean ± S.D.

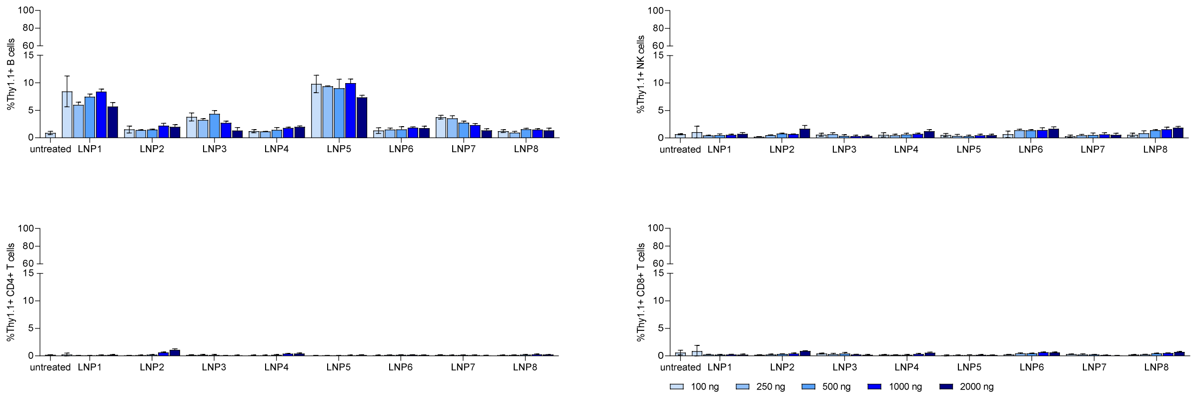

Figure S2. *In vitro* Thy1.1 expression in hPBMC sub-populations. Dose-range from 100 ng to 2000 ng. Transfection efficiency of LNP1-8 is shown in each sub-population. (**A**) %Thy1.1+ B cells, (**B**) %Thy1.1+ NK cells, (**C**) %Thy1.1 CD4+ T cells, (**D**) %Thy1.1+ CD8+ T cells. Data are presented as mean ± S.D., n= 3 technical replicates per LNP formulation.

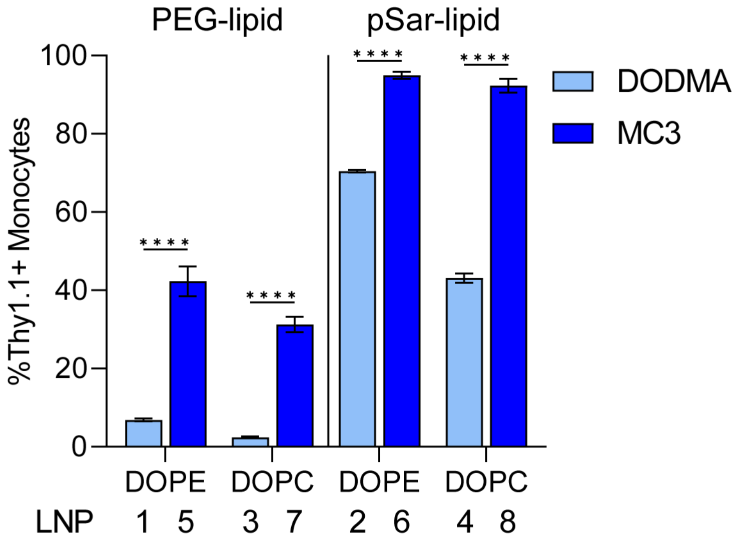

Figure S3. *In vitro* transfection efficiency of all DODMA-LNPs versus MC3-LNPs at a dose of 1000 ng. Transfection efficiency is shown as %Thy1.1+ Monocytes. Data are presented as mean ± S.D., analyzed by a two-way ANOVA with Šidák's multiple comparison test, ****P < 0.0001, n= 3 technical replicates per LNP formulation.

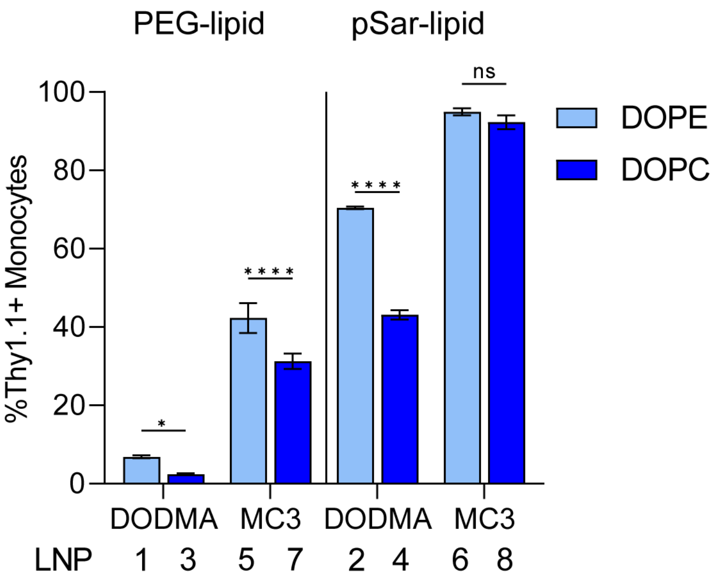

Figure S4. *In vitro* transfection efficiency of all DOPE-LNPs versus DOPC-LNPs at a dose of 1000 ng. Transfection efficiency is shown as %Thy1.1+ Monocytes. Data are presented as mean ± S.D., analyzed by a two-way ANOVA with Šidák's multiple comparison test, *P 0.0258, ****P < 0.0001, n= 3 technical replicates per LNP formulation.

Table S1. Results of peak fitting using Lorentzian function

	Manufacturing buffer						phosphate buffer pH 4.5					
Formu lation	area (a.u.)	σ	peak posi-tion (nm^{-1})	σ	width (nm^{-1})	σ	area (a.u.)	σ	Peak posi-tion (nm^{-1})	σ	width (nm^{-1})	Σ
LNP1	784.6	47.16	1.050	0.001	0.291	0.011	629.5	16.87	1.065	0.001	0.274	0.006
LNP2	756.5	83.51	1.034	0.002	0.435	0.029	571.6	15.02	1.064	0.001	0.286	0.006
LNP3	685.4	46.27	0.965	0.001	0.337	0.014	638.6	17.37	1.012	0.001	0.343	0.008
LNP4	744.2	55.95	0.950	0.003	0.445	0.021	655.6	20.51	1.002	0.002	0.346	0.009
LNP5	591.5	23.83	1.129	0.001	0.282	0.008	480.7	14.27	1.225	0.001	0.234	0.005
LNP6	566.9	17.13	1.083	0.001	0.273	0.006	452.9	11.08	1.215	0.001	0.225	0.005
LNP7	495.5	28.01	1.120	0.002	0.277	0.011	518.2	6.21	1.166	0.001	0.199	0.002
LNP8	452.6	19.94	1.079	0.001	0.272	0.009	498.5	9.41	1.170	0.001	0.198	0.003
